# Wild Felids as Hosts for Human Plague, Western United States

**DOI:** 10.3201/eid1512.090526

**Published:** 2009-12

**Authors:** Sarah N. Bevins, Jeff A. Tracey, Sam P. Franklin, Virginia L. Schmit, Martha L. MacMillan, Kenneth L. Gage, Martin E. Schriefer, Kenneth A. Logan, Linda L. Sweanor, Mat W. Alldredge, Caroline Krumm, Walter M. Boyce, Winston Vickers, Seth P.D. Riley, Lisa M. Lyren, Erin E. Boydston, Robert N. Fisher, Melody E. Roelke, Mo Salman, Kevin R. Crooks, Sue VandeWoude

**Affiliations:** Colorado State University, Fort Collins, Colorado, USA (S.N. Bevins, J.A. Tracey, S.P. Franklin, V.L. Schmit, M.L. MacMillan, L.L. Sweanor, C. Krumm, M. Salman, K.R. Crooks, S. VandeWoude); Centers for Disease Control and Prevention, Fort Collins (K.L. Gage, M.E. Schriefer); Colorado Division of Wildlife, Montrose, Colorado, USA (K.A. Logan, M.W. Alldredge); University of California, Davis, California, USA (W.M. Boyce, W. Vickers); National Park Service, Thousand Oaks, California, USA (S.P.D. Riley); United States Geological Survey, Irvine, California, USA (L.M. Lyren, E.E. Boydston, R. Fisher); National Cancer Institute, Bethesda, Maryland, USA (M.E. Roelke)

**Keywords:** Disease ecology, Lynx rufus, Puma concolor, Yersinia pestis, zoonoses, plague, bacteria, dispatch

## Abstract

Plague seroprevalence was estimated in populations of pumas and bobcats in the western United States. High levels of exposure in plague-endemic regions indicate the need to consider the ecology and pathobiology of plague in nondomestic felid hosts to better understand the role of these species in disease persistence and transmission.

Zoonotic pathogens account for ≈60% of emerging diseases (*1*,*2*). *Yersinia pestis*, a vector-borne bacterium and the causative agent of plague in mammals, is 1 such emergent pathogen (*3*). Plague is maintained among rodent hosts and their fleas; however, spillover into accidental hosts can result in severe illness and death, as well as geographic spread of the disease (*4*).

Domestic cats are a major source of human plague infections in the United States (*5*), putting veterinary workers and pet owners at risk for *Y. pestis* infections. During 1924–2006, a total of 13 human cases of primary pneumonic plague were documented in the United States, and >5 were associated with felids (D. Wong, pers. comm.). Twelve cases of plague transmission from nondomestic carnivores to humans have been documented (*5*–*7*), including a fatal case of human pneumonic plague in 2007 that resulted from direct contact with an infected puma (*Puma concolor*) (*8*). Despite the known association of felids with human plague, the prevalence of *Y. pestis* infection in nondomestic cats remains relatively unknown.

Pumas and bobcats (*Lynx rufus*) are 2 of the most widespread felids in North American, with pumas having the greatest range of any wild terrestrial mammal in the Western Hemisphere (*9*). Both species inhabit large territories and travel great distances during dispersal (*9*,*10*). These highly mobile animals may periodically reintroduce *Y. pestis*–positive fleas to distant regions, especially during epizootics (*11*). Consequently, carnivore-aided flea dispersal could play an important role in the spread and persistence of plague during interepizootic periods.

We examined plague exposure in populations of bobcats and pumas in California and Colorado. This gave us an opportunity to evaluate *Y. pestis* seroprevalence in multiple difficult-to-sample, plague-susceptible felid species across a wide geographic area.

## The Study

We collected samples from 119 pumas and 212 bobcats (Table 1) in 3 locations in southern California and 2 locations in western and north-central Colorado (Figure) from autumn 2002 through summer 2008. Seventy-seven of these bobcat samples consisted of thoracic fluid collected postmortem from hunter-killed animals. Eight puma samples collected in the 1980s served as historical reference for puma samples from the Colorado Western Slope (i.e., area west of the Continental Divide). Animals were captured, sampled, and released with permission of cooperating agencies after approval by animal care and use committees. Samples were processed according to protocol (*12*).

**Table 1:** Sample sizes for categorical variables, by location, in serosurvey for *Yersinia pestis* in wild felids, western United States, 2002–2008*

Category	Front Range, CO	Orange County, CA	San Diego/Riverside counties, CA	Ventura County, CA	Western Slope, CO	Mean seroprevalence (95% confidence interval)
Species						
Bobcat	0	73	0	61	78	13.77 (4.90–33.07)
Puma	33	5	38	4	36	8.17 (2.97–20.56)
Age						
Young (< 2 y)	5	23	5	29	45	6.70 (2.31–17.87)
Adult (>2 y)	27	49	27	31	68	16.52 (7.03–34.09)
Sex						
F	20	37	20	32	43	14.01 (5.65–30.70)
M	13	40	18	29	70	8.02 (3.01–19.69)
Season						
Fall	3	18	6	15	21	0
Spring	13	7	18	1	2	23.67 (11.27–43.09)
Summer	6	6	2	3	90	7.49 (0.79–45.06)
Winter	10	47	12	42	112	6.31 (2.74–13.88)

*All samples were serum samples, except for Western Slope bobcats, which were thoracic fluid samples.

**Figure:**
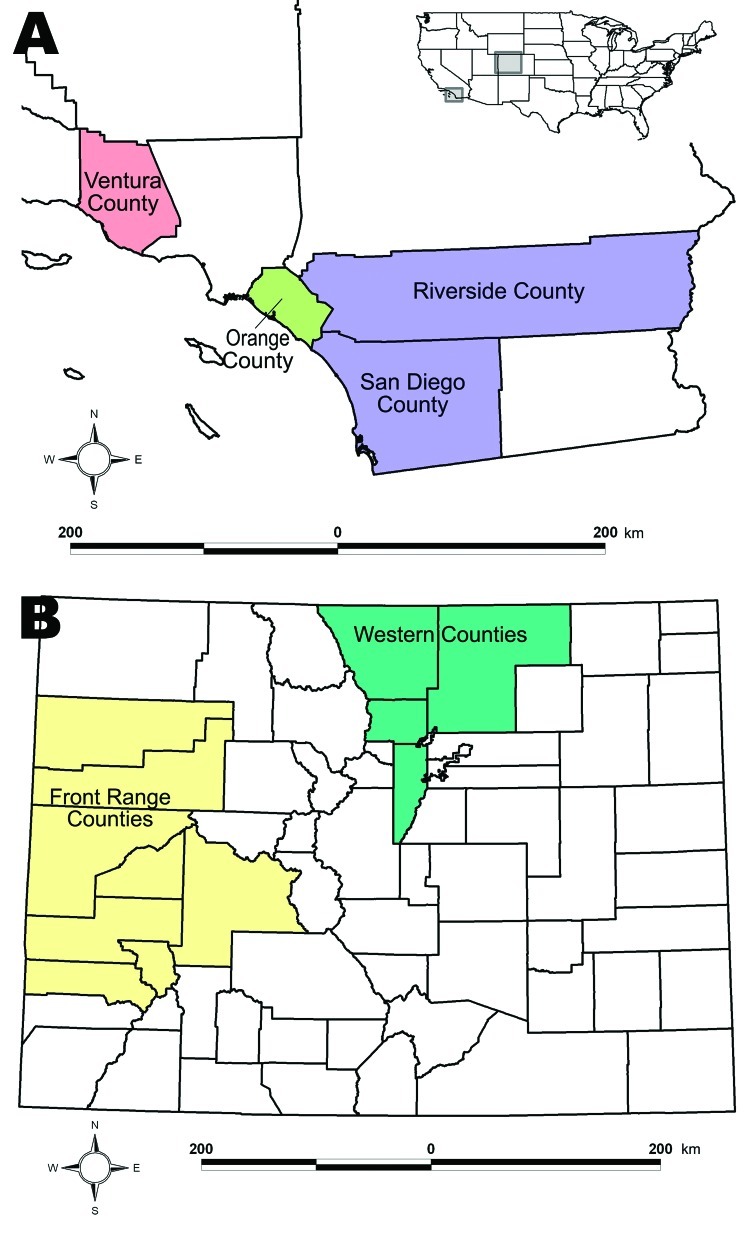
A) Study locations in California. B) Study locations in Colorado. Inset shows relative locations within the United States.

Thoracic fluid samples were immunoblotted onto nitrocellulose membranes (immuno-blot polyvinylidene fluoride membranes; Bio-Rad, Hercules, CA, USA) and probed with goat-anti-cat-phosphatase labeled antibody to verify the presence of immunoglobulin. Reacted membranes were rinsed 3 times with phosphate-buffered saline, once in Milli-Q (Millipore, Billerica, MA, USA) and were then exposed to a 5-bromo-4-chloro-3-indolyl-phosphate/nitroblue tetrazolium (alkaline-phosphatase chromogen) substrate (Kirkegaard and Perry Laboratories, Gaithersburg, MD, USA). Samples were classified by comparing staining intensity to positive (bobcat/domestic cat serum) and negative controls (water and goat serum).

Serum and thoracic fluid samples were analyzed for *Y. pestis* antibody using a hemagglutination assay according to a standard protocol (*13*). Positive samples were evaluated according to Chu (*13*). If a limited amount of sample was available, serum was diluted 1:4 and considered positive if titers were >32. Larger serum samples were not diluted, and a reading >16 was considered positive (*13*).

Data were analyzed by using a logistic link function and binary error, with antibody presence (positive vs. negative) as the outcome variable (SAS version 9.1; SAS, Cary, NC, USA). Estimates used maximum likelihood. Degrees of freedom were calculated by using a Kenward-Roger adjustment. Categorical factors included location, species, age, sex, and capture season. Animals captured in the fall (September–November) and in Ventura County were not plague positive and were omitted. All factors were treated as fixed variables, including location, because of previously reported differences in regional seroprevalence rates.

A total of 76 of 77 thoracic fluid samples had immunoglobulin present, as assessed by visual comparison of immunoblot staining, and were included in *Y. pestis* antibody analysis. Interactions were not significant and were omitted. Mean *Y. pestis* seroprevalence for pumas and bobcats across all locations was 17.7% (95% confidence interval [CI] 13.6%–21.8%). However, considerable variability existed across locations (Front Range, Colorado, mean 21.1 [95% CI 8.23–44.75]; Orange County, California, mean 1.23 [95% CI 0.13–10.01]; San Diego/Riverside counties, California, mean 6.58 [95% CI 1.52–24.33]; Ventura County, California, mean 0 [NA]; Western Slope, Colorado, mean 46.03 [95% CI 24.37–69.29]). Species and sex were not significant predictors of plague exposure; however, animal age, geographic location, and capture season were significant (Table 2). Adult animals (>2 years of age) and animals from the Colorado Western Slope were more likely to be seropositive (Table 1). Sixty-three percent (5/8) of historical puma samples from the Western Slope had detectable plague antibodies, similar to the seroprevalence rate of contemporary puma samples from this region (46.03%). Season also played a role, and spring-captured animals were more likely to be seropositive (Tables 1 and 2).

**Table 2:** Potential fixed-effect predictors of plague exposure in pumas and bobcats, western United States, 2002–2008*

Fixed effect	Num df	Den df	F value	p value
Age	1	287	5.13	**0.024**
Location	4	287	8.36	**<0.0001**
Season	3	287	4.1	**0.0179**
Sex	1	287	2.47	0.117
Species	1	287	1.02	0.314

*Num df, numerator degrees of freedom; den df, denominator degrees of freedom. **Boldface** indicates significance (p<0.05).

Colorado sample sites showed 51 (38%) positive of 135 animals tested. Seroprevalence rates in the Colorado sample areas were 21% (Front Range) and 46% (Western Slope) respectively, a higher proportion than expected given the severe disease seen in plague infections in some domestic cats (*3*). California sample sites had limited plague seroreactivity, with only 4 (2.2%) of 181 animals positive for plague exposure.

The Colorado Western Slope is near the Four Corners region (i.e., contiguous boundaries of southwestern Colorado, northwestern New Mexico, northeastern Arizona, and southeastern Utah). During 1957–2004, a total of 419 human plague cases were documented in the United States, of which 83% were from this region (*14*). The complex dynamics governing high plague incidence in this region are not fully understood despite extensive research but most likely involve climate, mammalian reservoirs, vector species, and habitat ecotypes (*4*,*7*,*14*).

## Conclusions

Plague dynamics often are characterized by epizootics, resulting in interannual variation in infection rates among plague hosts; however, seroprevalence of 8 puma samples collected in the 1980s mirrored contemporary samples collected since 2002 and may indicate high levels of sustained plague activity in the area in this species. Seroprevalence rates were similar across multiple sample years. Vector-borne disease often is highly seasonal because of annual shifts in vector activities and abundance (*4*); however, seasonal patterns based on serologic data must be interpreted with caution because of long-term antibody persistence in some recaptured animals (S.N. Bevins, unpub. data).

Puma and bobcat data from this study suggest exposure followed by recovery. All animals were outwardly healthy. Deaths caused by plague have been documented in wild felids (*8*,*9*,*15*), and the potential for plague exposure remains a concern for field biologists, veterinarians, hunters, and skinners. Field biosafety guidelines have been developed in conjunction with Colorado State University’s Biosafety Office as a result of these findings. Recommendations include wearing disposable gloves, long pants, and long-sleeved shirts when handling anesthetized animals and using an N95-rated mask when conducting necropsies or handling deceased animals. Outside of human infections, plague could constitute a problem for felid conservation in areas of high plague activity (*1*,*15*).

Results suggest large numbers of *Y. pestis*–exposed pumas and bobcats. Regular serosurveys that document seroreactivity increases above an original baseline could indicate epizootic activity in felids and other plague hosts. High regional seroprevalence indicate these animals may be involved in the persistence and transmission of *Y. pestis*. This and the documented transmission of plague from nondomestic carnivores to humans (*6*–*8*) emphasize the need to better understand the role of wild felids in plague dynamics.
